# Pre-neuromusculoskeletal injury Risk factor Evaluation and Post-neuromusculoskeletal injury Assessment for Return-to-duty/activity Enhancement (PREPARE) in military service members: a prospective, observational study protocol

**DOI:** 10.1186/s12967-022-03832-7

**Published:** 2022-12-25

**Authors:** Courtney M. Butowicz, Brad D. Hendershot, Nora L. Watson, Daniel I. Brooks, Donald L. Goss, Robert A. Whitehurst, Alisha D. Harvey, Matthew S. Helton, Joseph R. Kardouni, Matthew B. Garber, Timothy C. Mauntel

**Affiliations:** 1 Research & Surveillance Division, Extremity Trauma and Amputation Center of Excellence, 4494 Palmer Rd N, Bethesda, MD 20814 USA; 2 Research & Development Section, Department of Rehabilitation, Walter Reed National Military Medical Center, 4494 Palmer Rd N, Bethesda, MD 20814 USA; 3 Department of Physical Medicine & Rehabilitation, Uniformed Services University of the Health Sciences, 4301 Jones Bridge Rd, Bethesda, MD 20814 USA; 4 Department of Research, Walter Reed National Military Medical Center, 4494 Palmer Rd N, Bethesda, MD 20814 USA; 5 Clinical Quality Management, Defense Health Agency, 7700 Arlington Blfd, Falls Church, VA 22042 USA; 6 Department of Physical Therapy, High Point University, 1 N University Pkwy, High Point, NC 27268 USA; 7 U.S. Army Forces Command, 4700 Knox St, Fort Bragg, NC 28301 USA; 8 Physical Therapy Service, Department of Rehabilitation, Walter Reed National Military Medical Center, 4494 Palmer Rd N, Bethesda, MD 20814 USA; 9 Department of Health, Human Function and Rehabilitation Science, The George Washington University, 2200 Pennsylvania Ave NW, Washington, DC, 20006 USA; 10 Research & Surveillance Divsion, Extremity Trauma & Amputation Center of Excellence, 2817 Reilly Rd, Fort Bragg, NC 28310 USA; 11 Department of Surgery, Uniformed Services University of the Health Sciences, 4301 Jones Bridge Rd, Bethesda, MD 20814 USA; 12 Department of Clinical Investigations, Womack Army Medical Center, 2817 Reilly Rd, Fort Bragg, NC 28301 USA

**Keywords:** Musculoskeletal injury, Neuromuscular, Military, Service member, Tactical athlete, Risk assessment, Risk mitigation

## Abstract

**Background:**

Non-battle related musculoskeletal injuries (MSKI) are one of the primary medical issues diminishing Service member medical readiness. The MSKI problem is challenging because it is difficult to assess all of the factors that increase MSKI risk and influence post-MSKI outcomes. Currently, there are no high-throughput, clinically-feasible, and comprehensive assessments to generate patient-centric data for informing pre- and post-MSKI risk assessment and mitigation strategies. The objective of the “Pre-neuromusculoskeletal injury Risk factor Evaluation and Post-neuromusculoskeletal injury Assessment for Return-to-duty/activity Enhancement (PREPARE)” study is to develop a comprehensive suite of clinical assessments to identify the patient-specific factors contributing to MSKI risks and undesired post-MSKI outcomes.

**Methods:**

This is a phased approach, multi-center prospective, observational study (ClinicalTrials.gov number: NCT05111925) to identify physical and psychosocial factors contributing to greater MSKI risk and undesired post-MSKI outcomes, and to identify and validate a minimal set of assessments to personalize risk mitigation and rehabilitation strategies. In Phase I, one cohort (n = 560) will identify the physical and psychosocial factors contributing to greater MSKI risks (single assessment), while a second cohort (n = 780) will identify the post-MSKI physical and psychosocial factors contributing to undesired post-MSKI outcomes (serial assessments at enrollment, 4 weeks post-enrollment, 12 weeks post-enrollment). All participants will complete comprehensive movement assessments captured via a semi-automated markerless motion capture system and instrumented walkway, joint range of motion assessments, psychosocial measures, and self-reported physical fitness performance and MSKI history. We will follow participants for 6 months. We will identify the minimum set of clinical assessments that provide requisite data to personalize MSKI risk mitigation and rehabilitation strategies, and in Phase II validate our optimized assessments in new cohorts.

**Discussion:**

The results of this investigation will provide clinically relevant data to efficiently inform MSKI risk mitigation and rehabilitation programs, thereby helping to advance medical care and retain Service members on active duty status.

*Trial Registration*: PREPARE was prospectively registered on ClinicalTrials.gov (NCT05111925) on 5 NOV 2021, prior to study commencement.

## Background

Musculoskeletal injuries (MSKI) are endemic among military Service members and are the leading cause of limited/lost duty time [1, 2]. It is critical we identify the factors contributing to non-battle related MSKI risks and post-MSKI outcomes such that targeted MSKI risk mitigation and rehabilitation programs may be developed. A comprehensive biopsychosocial model is required to identify the pre- and post-MSKI physical and psychosocial factors that contribute to future MSKI risks and post-MSKI outcomes [3].

Service member specific MSKI risk factors and predictors of post-MSKI outcomes are multifaceted and interrelated. The primary predictor of future MSKI is previous MSKI [4, 5]. Additionally, aberrant movement patterns identify MSKI risks and may contribute to the development and/or recurrence of musculoskeletal conditions, such as low back pain [3, 6, 7]. Pre- and post-MSKI aberrant movement patterns may be exacerbated by low physical fitness [7], which in itself is an MSKI risk factor [4, 5, 7, 8]. Furthermore, sub-optimal joint mobility (e.g., restricted range of motion, capsular tightness) can contribute to aberrant movement patterns [9, 10] and potentially increase MSKI risks [11–13]. A number of potential post-MSKI physical [14–17] as well as deleterious psychosocial factors (e.g., fear of movement) may negatively impact sensorimotor function and movement quality, thereby increasing MSKI risks [18, 19] and exacerbating musculoskeletal conditions [3]. While the aforementioned factors are potential risk factors for subsequent MSKI, it is also possible the risk factors preceding the initial MSKI may not have been addressed or resolved with rehabilitation and therefore may still pose a risk for future MSKI.

Field-expedient movement assessments can efficiently identify individuals at greater MSKI risk within clinical settings [4–6, 20–22]. However, clinicians must utilize field-expedient functional assessments of varying difficulty and intensity to comprehensively identify movement related MSKI risks [23]. Common lower extremity movement assessments include the double and single leg squat [9, 10], jump-landings [20], and the triple hop for distance [24]. These assessments collectively evaluate lower extremity strength and neuromuscular control, and can identify inter-limb asymmetries; a key MSKI risk factor and predictor of post-MSKI outcomes [24]. Additionally, multivariate spatiotemporal gait assessments can quantify functional capabilities in physically active individuals, which is particularly relevant to Service members who often perform long distance ruck marches [16]. Common upper extremity movement assessments include the Closed Kinetic Chain Upper Extremity Stability Test [21, 22] and the Functional Movement Screen (FMS) Shoulder Clearing Test [4, 5]. Of note, individual FMS items are better MSKI risk predictors than cumulative FMS scores [4, 5, 23]. Similar to extremity MSKI risk factor evaluations, field-expedient assessments including the prone plank and the Active Hip Abduction [25] assessments are recommended for clinical use to evaluate low back pain risk [26].

Biopsychosocial approaches are required to comprehensively identify all of the factors that affect MSKI risks and outcomes [3]. The National Institutes of Health (NIH) Patient Reported Outcomes Measurement Information System (PROMIS) measures common domains (e.g., depression, physical function) associated with MSKI risk across populations [27]. Previous work [16], demonstrates PROMIS measures can detect changes in self-reported physical function following MSKI [27]. Furthermore, pain-related fear of movement (i.e., kinesiophobia) [28] is associated with limb asymmetries and stiffer movement patterns during activities, which may increase MSKI risks [19], and it is a determinant of whether individuals will successfully return-to-duty/activity (RTD/A) after MSKI [18, 19, 29].

The greatest limitations to previously proposed comprehensive MSKI assessments are clinical skills/expertise and time. Semi-automated motion capture systems can reduce the technical expertise required to successfully complete movement assessments and to help expedite the assessment process. Semi-automated motion capture systems have rigid objectivity that reduces inter-rater subjectivity [30] and improves the clinical utility of field-expedient functional assessments [23]. The semi-automated nature of these systems provides objective data and removes the significant time barriers (training and scoring) required for many field-expedient assessments when they are completed en masse (e.g., a company or battalion) [30]. Similarly, instrumented walkways efficiently provide valid measures of common gait variables [31] and can quantify triple hop distances [24]. Additionally, PROMIS computer adaptive testing (CAT) formats reduce question burden while increasing precision [27]. All of these systems require less than 15 min to train individuals how to operate and require no more time for data analyses than it takes for the individual to complete the assessments, making them ideal for clinical settings and novice raters.

The primary objective of the “Pre-neuromusculoskeletal injury Risk factor Evaluation and Post-neuromusculoskeletal injury Assessment for Return-to-duty/activity Enhancement (PREPARE)” study is to develop a high-throughput comprehensive suite of clinical assessments that collectively identify the Service member-specific factors that contribute to MSKI risks and undesired post-MSKI outcomes. Furthermore, we aim to develop and validate optimized versions of our pre- and post-MSKI assessments that efficiently identify the physical and psychosocial factors that inform MSKI risk mitigation and rehabilitation strategies. We hypothesize a set of field-expedient assessments can efficiently identify Service member-specific MSKI risk factors, post-MSKI deficits, and provide data to guide future patient-specific risk mitigation and rehabilitation programs.

## Methods/design

### Study design

**Fig. 1:**
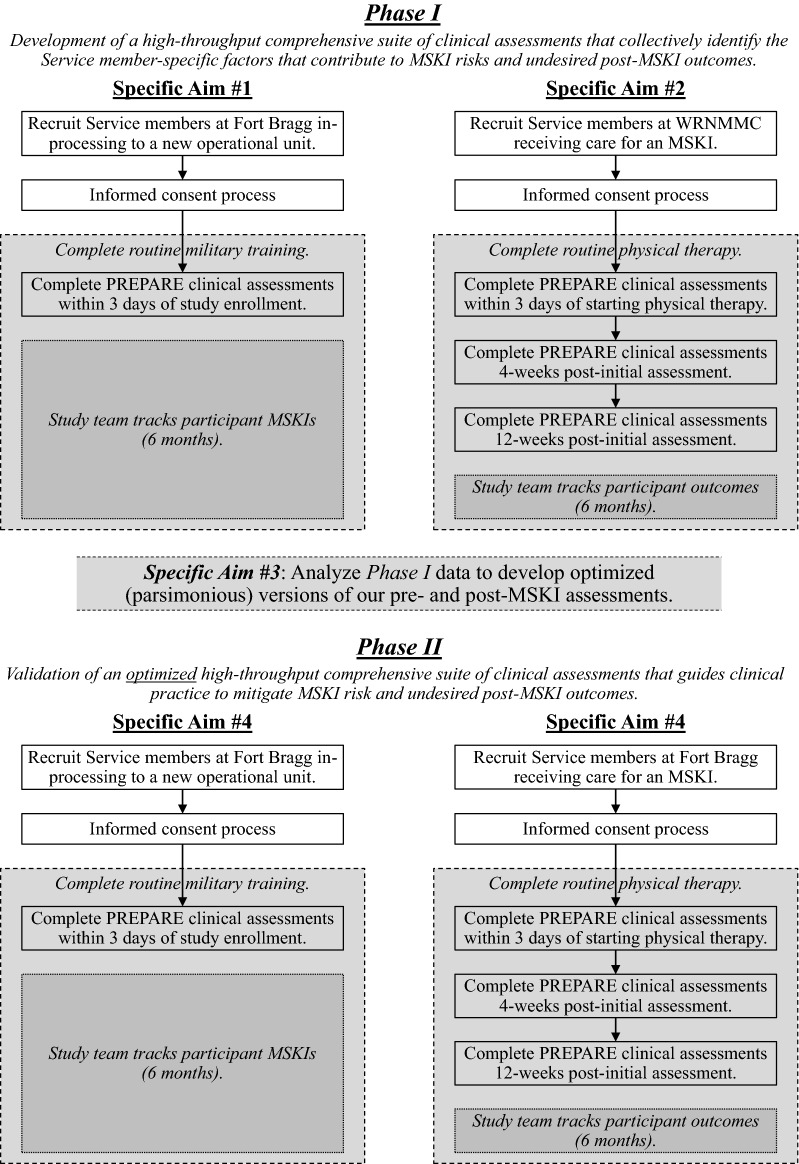
Study flow diagram

This is a multi-center, prospective, observational study to create a suite of clinical assessments that collectively identify the patient-specific factors contributing to MSKI risks and undesired post-MSKI outcomes. We will utilize a phased approach to complete our objectives: Phase I will evaluate the physical and psychosocial factors contributing to greater MSKI risks and undesired post-MSKI outcomes, and identify the minimum set of assessments that provide the data needed to personalize MSKI risk mitigation and rehabilitation strategies; Phase II will validate these optimized MSKI clinical assessments in newly enrolled cohorts. Figure 1 provides the study flow of our investigation. This study design will allow us to answer our specific aims:

*Specific Aim #1*: Determine the physical and psychosocial factors that differentiate between Service members who do vs. do not sustain a MSKI within 6 months of study enrollment. *Hypothesis: Semi-automated field-expedient assessments can identify the factors that differ between Service members who do vs. do not sustain a MSKI.*

*Specific Aim #2*: Determine the post-MSKI physical and psychosocial factors that differentiate between Service members with conservatively managed MSKIs who do vs. do not develop undesired outcomes within 6 months of study enrollment. *Hypothesis: Semi-automated field-expedient assessments can identify the factors that differ between Service members who do vs. do not develop undesired post-MSKI outcomes.*

*Specific Aim #3*: Develop optimized versions of both pre- and post-MSKI assessments that efficiently identify the physical and psychosocial factors that inform MSKI risk mitigation and rehabilitation strategies. *Hypothesis: A common set of semi-automated field-expedient assessments can inform clinical decision-making regarding which Service members are at the highest risk of sustaining MSKIs and undesired post-MSKI outcomes.*

*Specific Aim #4*: Validate the optimized versions of both pre- and post-MSKI assessments by demonstrating their abilities to predict MSKI risks and outcomes in new Service member cohorts. *Hypothesis*: *The optimized pre- and post-MSKI assessments will be able to accurately identify Service members at the highest risk for sustaining MSKIs and undesired post-MSKI outcomes across Service member populations*.

### Ethical aspects

This study was approved by the Walter Reed National Military Medical Center (WRNMMC) Institutional Review Board (protocol WRNMMC-2020-0283) and registered with ClinicalTrials.gov (NCT05111925). The Fort Bragg performance site relied on the WRNMMC Institutional Review Board for study protocol review and regulatory oversight. Any changes to the study protocol will be requested through the WRNMMC Institutional Review Board and reflected on ClinicalTrials.gov.

Study participants will sign a site-specific written informed consent form, prior to completing study-related tasks. Upon enrollment in the study, study participants will be randomly assigned a unique participant identification number. Participants will only be referred to in study documentation by their unique identification number and the same number will be used across all data collection systems. Coded data will be stored on password-protected, encrypted networks and access to participant data will be limited to the study team and other authorized entities.

### Study population

A total of 2,680 active duty Service members between 18 and 44 years of age will be enrolled in this study. In Phase I and Phase II we will recruit convenience samples of active duty Service members in-processing to new military units at Fort Bragg, NC. Participants will be identified during their in-processing medical screenings. Additionally, in Phase I we will recruit a convenience sample of active duty Service members receiving physical therapy for a MSKI at WRNMMC. In Phase II, we will recruit a convenience sample of active duty Service members receiving physical therapy for a MSKI at Fort Bragg, NC. In both Phase I and Phase II, participants with a MSKI will be identified by their physical therapists during their initial encounters.

### Inclusion and exclusion criteria

The study inclusion and exclusion criteria will determine if potential study participants are eligible to participate in the study (Table 1). Inclusion and exclusion criteria will be self-reported by the potential participants, and study team members will not access the participant’s medical record or incorporate pregnancy testing to confirm inclusion/exclusion criteria.

**Table 1:** Participant inclusion and exclusion criteria

Cohort	Inclusion criteria	Exclusion criteria
Fort Bragg—In-processing (Specific Aim #1; Specific Aim #4)	• Active duty service members in-processing to a unit at Fort Bragg, NC • Recruited ≤ 14 days following in-processing medical screening • 18–44 years of age^a^	• On limited duty status for any reason • Scheduled for a deployment or separation within 12 months • Pregnant^b^
WRNMMC—Injured (Specific Aim #2)	• Active duty service member receiving conservative care for a lower extremity or low back MSKI • Recruited ≤ 3 days following presentation to the physical therapy clinic for care of the index MSKI • 18–44 years of age^a^	• Musculoskeletal related surgery • MSKI within the previous 6 months that resulted in altered/missed physical activity for ≥ 3 consecutive days • Ongoing TBI related issues^c^ • Pregnant^b^
Fort Bragg—Injured (Specific Aim #4)		

^a^18–44 years old accounts for 86% of deployed individuals[42]

^b^pregnant females will be eligible for participation after the pregnancy and medical clearance by a healthcare provider

^c^TBI (traumatic brain injury) is a recognized MSKI risk factor[43]

### Data collection

Given the goal of this study to develop field-expedient and clinic-friendly solutions to quantify metrics from functional assessments, we will use a semi-automated commercially available markerless motion capture system and instrumented walkway. The HumanTrak (VALD Health, Charlotte, NC) markerless motion capture system will quantify/estimate joint kinematics during functional assessments (i.e., double leg squats, single-leg squats, jump-landings) [32]. The Zeno Walkway (ProtoKinetics LLS.; Havertown, PA) will efficiently provide valid measures of common gait variables [31] and quantify triple hop distances [24]. Range of motion data will be measured with standard clinical goniometers and digital inclinometers. Participant reported data will be collected electronically via a standardized research data collection platform.

### Phase I approach

We will concurrently enroll cohorts for two separate observational studies: one at Fort Bragg, NC (n = 560) and one at WRNMMC (n = 780). The Fort Bragg cohort will identify the physical and psychosocial factors that contribute to greater MSKI risks (Specific Aim #1), while the WRNMMC cohort will identify the post-MSKI physical and psychosocial factors that contribute to undesired participant outcomes (Table 4, Specific Aim #2). Participants enrolled in Specific Aim #1 who go on to sustain an MSKI during the study follow-up period will be invited to participate into Specific Aim #2, and thus undergo post-MSKI tracking/assessments. Both cohorts will complete identical testing procedures. The study team will track study-related outcomes and pull relevant health related information through the Military Health System Data Repository (MDR) and participant self-report data. The MDR contains records on all healthcare events paid for by the Military Health System and is the best source of Military Health System data available for researchers.

#### Clinical assessments

For Specific Aim #1*,* all clinical assessments will be collected during a single testing session at the time of study enrollment. For Specific Aim #2, participants will undergo repeat (≤ 3 days of starting physical therapy [“initial”]; 4 weeks post-initial assessment, or at RTD/A clearance [i.e., cleared from physical therapy], if prior to 4 weeks; and 12 weeks post-initial assessment, or at RTD/A clearance, if prior to 12 weeks) clinical assessments.

#### Functional assessments

Lower extremity movement assessments will be recorded and automatically quantified using the markerless motion capture system or instrumented walkway. All participants will complete the assessments in the same order, progressing from less to more intense assessments. Upper extremity assessments will follow lower extremity assessments. Testing order will follow the order described in Table 2.

**Table 2:** Functional movement assessments and outcomes

Assessment	Equipment	Description	Outcome measure(s)
Gait [16] *Trials: all recorded during 2 min walk test*	Instrumented walkway	Participants will complete over-ground gait trials at a self-selected pace during a 2 Min Walk Test [44]. Participants will be instructed to minimize “targeting” and a trial will be recorded each time the participant walks across the walkway	Spatiotemporal parameters, including: • Gait velocity; • Single and double limb support time; • Measures of variability for stride time and length, and step length, time, and width
Double-leg squat [10] *Trials: 3 sets of 3* *(9 total)*	Markerless motion capture	Participants will stand with their feet shoulder-width apart, feet pointed anteriorly, heels on the floor, and arms extended overhead. Participants will squat in a controlled manner, to a comfortable depth	Trunk and lower extremity kinematics
Single-leg squat [9] *Trials: 3 sets of 3 per limb* *(18 total)*	Markerless motion capture	Participants will stand on the test limb, with the foot pointed anteriorly, heel on the floor, hands on their hips, and head facing anteriorly. The non-weight bearing limb will be flexed to 90˚ of hip and knee flexion. Participants will squat in a controlled manner, to a comfortable depth, and so they can maintain their balance	Trunk and lower extremity kinematics
Jump-landing [20] *Trials: 3*	Markerless motion capture	The participant will stand on a 30 cm tall box, set 3 feet behind a target area. Participants will jump out, not vertically, to reach the target area with both feet leaving the box at the same time; upon landing the participant will immediately jump vertically for maximal height	Trunk and lower extremity kinematics
Triple hop [24] *Trials: 3 per limb* *(6 total)*	Instrumented walkway	Participants will stand with their heel on the edge of the instrumented walkway. Participants will perform three consecutive maximal forward hops on the same limb, “sticking the landing” on the last hop	Average distance covered across trials for each limb and limb symmetry index [33]
Closed kinetic chain upper extremity stability test [21] *Trials: 3*	Stopwatch, athletic tape, tape measure	Participants will assume a push-up position with each hand placed on a mark spaced 36 inches apart with the shoulders aligned over the hands. As quickly as possible, participants will: (1) lift one hand and touch the opposite mark, (2) replace the original hand on the original mark, (3) repeat this process on the opposite hand, and then (4) continue for 15 s	Each trial will be examined 3 ways and averaged across trials: • Number of touches; • Number of touches normalized to participant height; • “Power score”, calculated by multiplying the number of touches by 68% of the participant’s mass then dividing by 15 s
FMS Shoulder clearing test [4, 5] *Trials: 1 per limb*	None	Participants will place their hand on the opposite shoulder and point the elbow upward	Participants will be dichotomized as having pain or not; results recorded bilaterally
Prone plank assessment [45] *Trials: 1*	Stopwatch	Participants will lie prone with the elbows and hands parallel to each other and in contact with the ground, so the humeri are aligned beneath the shoulders. Participants will maintain a rigid body position as long as possible so that the entire body weight is supported by the elbows and toes, while the ankles, knees, hips, and spine maintain a neutral alignment	Total time
Active hip abduction [25] *Trials: 1 per limb*	None	Participants will lie on their sides with the pelvis and lower extremities in line with the trunk and then abduct the hip, attempting to keep the knee extended and lower limb aligned with the trunk	Trials will be rated on an ordinal scale of 0, 1, 2, or 3. The lower of the two scores will be used for the final overall score

#### Range of motion assessments

The joint will be moved through its range of motion (Table 3) to the point of first resistance or until the participant indicates discomfort. Three trials of each assessment will be recorded bilaterally, alternating limbs between measurements. To optimize efficiency, all participants will complete the range of motion assessments in the same order. Measurements will be averaged across trials for each limb and limb symmetry indices will be calculated [33]. We will establish our team’s reliability for all measures, prior to study commencement.

**Table 3:** Range of motion assessments

Measurement	Testing procedures	
Knee extension [9]	Supine with the test limb flexed to 90˚ of hip and knee flexion; the non-test limb extended on the testing table	An inclinometer placed just distal to the test limb tibial tuberosity; the angle of the tibia will be measured relative to the horizontal
Hip abduction [9]	Supine with the test and non-test limbs extended on the testing table	The goniometer stationary arm placed across the anterior superior iliac spines with the axis over the test limb anterior superior iliac spine; the movement arm will vertically bisect the participant’s thigh and move with the thigh
Hip internal rotation [9]	Prone with the test limb flexed to 90˚ of knee flexion; the non-test limb extended on the testing table	An inclinometer placed just proximal to the test limb lateral malleolus; the angle of the fibula will be measured relative to the vertical
Hip external rotation [9]	Prone with the test limb flexed to 90˚ of knee flexion; the non-test limb extended on the testing table	An inclinometer placed just proximal to the test limb lateral malleolus; the angle of the fibula will be measured relative to the vertical
Ankle dorsiflexion [46]	Standing with the test foot perpendicular to the wall; the non-test foot placed behind the test foot. The participant will lunge forward until right before the heel lifts off the floor	An inclinometer placed just distal to the test limb tibial tuberosity; the angle of the tibia will be measured relative to vertical

#### Participant reported outcomes

Participants will complete initial and follow-up (monthly) questionnaires. All questionnaires will be administered electronically, such that participants will be able to complete them from any location with internet access.

NIH PROMIS—we will utilize NIH PROMIS CAT measures for Physical Function, Pain Interference, Depression, and Anxiety. PROMIS measures will be converted to t-scores and scores that reach a floor or ceiling threshold (“outliers”) will be excluded from analyses.

Tampa Scale of Kinesiophobia [28]—we will utilize the abbreviated 11 question TSK-11. The total score will be calculated (summed) [28].

Musculoskeletal Injuries***—***participants will report previous MSKIs sustained within 1 year prior to study enrollment, any “severe” MSKIs they sustained that resulted in three or more consecutive weeks of missed activity, and any musculoskeletal surgeries. Participants will also report any new MSKIs they sustain throughout the study follow-up period. The questionnaire contains details regarding activity at the time of MSKI, the mechanism of injury, date of MSKI, and the geographic location.

Physical Fitness Test Performance***—***participants will report the scores from their most recent standard military physical fitness assessment. Raw scores will be converted to the standardized point system, based on Service specific requirements.

### Outcome measures

All study outcomes will be tracked for up to 6 months. The primary outcomes are if a Service member sustains a MSKI or experiences an undesired post-MSKI outcome. A MSKI will be defined as a medical encounter that is associated with a relevant International Classification of Diseases (ICD)-10 code [34, 35]. We will abstract the following data for each MSKI: (1) body part; (2) activity at the time of MSKI; (3) mechanism of injury; (4) date; and (5) geographic location. MSKIs that result from a contact mechanism or enemy combatants will be excluded from analyses. Post-MSKI outcomes will be dichotomized as either “desired” or “undesired” as described in Table 4. Participants with any “undesired” outcome will be considered to have an overall undesired outcome. The secondary outcomes include: (1) time until the index MSKI relative to study enrollment; (2) time to RTD/A following the index MSKI; and (3) healthcare utilization (e.g., physical therapy encounters).

**Table 4:** Participant post-musculoskeletal injury outcome classifications

Patient outcome	Operational definition	
	Desired (“Positive”) outcome	Undesired (“Poor”) outcome
Recurrent MSKI	No new MSKI within 6 months of RTD/A following cessation of treatment for the index MSKI	Same MSKI at the same location as the index MSKI within 6 months of RTD/A following cessation of treatment for the index MSKI
Subsequent MSKI condition	No new MSKI within 6 months of RTD/A following cessation of treatment for the index MSKI	A new MSKI at a different location as the index MSKI within 6 months of RTD/A following cessation of treatment for the index MSKI
Chronification [47]	Index MSKI signs/symptoms resolve within 12 weeks	Index MSKI signs/symptoms do not resolve within 12 weeks
Delayed RTD/A	RTD/A prior to 2 standard deviations greater than the mean number of limited duty days for our total sample	RTD/A more than 2 standard deviations greater than or equal to the mean number of limited duty days for our total sample
PROMIS physical function	Physical Function score is greater than 1 standard deviation less than the mean Physical Function score for our total sample at the 12 week follow-up for the index MSKI	Physical Function score is less than or equal to 1 standard deviation less than the mean Physical Function score for our total sample at the 12 week time point for the index MSKI
PROMIS pain interference	Pain Interference score is less than 1 standard deviation greater than the mean Pain Interference score for our total sample at the 12 week follow-up for the index MSKI	Pain Interference score is greater than or equal to 1 standard deviation greater than the mean Pain Interference score for our total sample at the 12 week follow-up for the index MSKI
TSK-11	TSK-11 score is less than 1 standard deviation greater than the mean TSK-11 score for our total sample at the 12 week follow-up for the index MSKI	TSK-11 score is greater than or equal to 1 standard deviation greater than the mean TSK-11 score for our total sample at the 12 week follow-up for the index MSKI

^*^Index MSKI = Qualifying MSKI that the participant experienced for enrollment into the study

### Phase I statistical and data analysis plan

Phase I analyses will extend a statistical approach previously shown to (1) identify distinct clusters of physical assessments representing meaningful domains of knee function, and (2) select optimal test batteries found to discriminate injured individuals versus healthy controls [36]. We will use a similar empirical strategy to reduce redundancy among our comprehensive assessments for MSKI-related risk. Descriptive analyses with traditional bivariate tests will evaluate score distributions overall and by incident MSKI and undesired outcomes. We will use hierarchical cluster analysis, a machine learning algorithm useful for representing natural groupings among observations, to next identify clusters of related continuous variables in the Phase I dataset. Distances between pairs of variables will be defined based on their correlations. Tree diagrams obtained from cluster analysis of the distance matrix will be used to identify variable clusters that will be interpreted based on current knowledge of underlying functional domains. Sensitivity analyses will assess the robustness of groupings to the choice of linkage method. Stratified analyses among the injured cohort will describe cluster reliability among subgroups defined by MSKI type (back pain vs extremity) at enrollment.

We will next define reduced, candidate test batteries as combinations of individual variables found to characterize distinct clusters representing meaningful functional domains [36]. Cluster representatives will be defined to maximize the clinical feasibility of the potential test battery, e.g., by minimizing estimated completion times. Candidate test batteries will be evaluated for each cohort in separate logistic regression models with component test scores as predictors and incident MSKI or undesired outcome (Table 4) as the dependent variable. Misclassification rates will be reported as the percent of participants where the outcome was incorrectly predicted after thresholding using clinically relevant sensitivities and specificities, with cross-validation to minimize bias in estimated probabilities. Models with the lowest model-based misclassification rates will be selected as optimal pre- and post-MSKI assessments and further evaluated in logistic regression models adjusted for age, sex, body mass index, physical fitness, history and duration of previous MSKI, and self-report of pain. Regression estimates and area under the curve (AUC) will be reported for adjusted and unadjusted models to describe predictive values of optimal assessments independent of traditional risk factors. Additional analyses will repeat final models defining MSKI by type (back pain vs extremity) to assess for changes in model performance with narrowed case definitions. Exploratory analyses will use regression-based variable selection methods (e.g., LASSO) to describe the potential predictive accuracy of available assessments while ignoring requirements for test feasibility or interpretation based on known functional domains.

#### Missing data

We expect infrequent (< 5%) missing functional measures at the baseline assessment based on completion rates in our previous work; however, multiple imputation will be considered if missingness exceeds 10% [37]. Because outcomes will be ascertained using the MDR, minimizing risk of loss to follow-up, no imputation of missing outcomes is planned.

#### Sample size

We assume event rates of 50% for incident MSKI (uninjured cohorts) [38] and 30% for undesired outcomes (injured cohorts) [39, 40], and 10% loss to follow over 6 months based on reported annual changes in beneficiary status in the MDR. We expect similar frequencies of back pain and extremity injuries based on previous ICD reporting of these MSKI types at our clinical sites. Targeted enrollment will be 560 Service members in the uninjured cohort (Specific Aim #1) and 780 Service members in the injured cohort (Specific Aim #2) to observe  > 100–125 outcomes in each cohort for each MSKI type. These expected event counts will yield  > 10 events per predictor variable in binary classification models with 10–12 predictors, allowing analyses to evaluate the predictive value of reduced assessments independent of traditional risk factors.

### Phase II approach

We will enroll active duty Service members who are in-processing to a new unit at Fort Bragg, NC (n = 560) and active duty Service members receiving physical therapy for a MSKI within a military physical therapy clinic (n = 780) into two new observational cohorts. We will validate the abilities of our optimal pre- and post-MSKI assessments to correctly identify Service members who are at high risk for MSKI or undesired post-MSKI outcomes (Specific Aim #4*)*. Identical to Phase I, participants who are enrolled into the uninjured cohort who go on to sustain a MSKI during the study follow-up period will be invited to participate in the injured cohort post-MSKI tracking/assessments.

#### Clinical assessments

We will administer our reduced battery of pre- and post-MSKI assessments (Specific Aim #3) among the newly enrolled validation cohorts. We will use the same data collection methods described for Phase I for the relevant assessments.

### Phase II statistical and data analysis plan

Phase II analyses will evaluate the ability of the reduced batteries to correctly predict incident MSKI and undesired outcomes in validation cohorts. Misclassification rates for unadjusted and full models will be described with the AUC and sensitivities and specificities based on relevant thresholds. Calibration will be plotted using locally estimated scatterplot smoothing (LOESS) smoothing of the predicted versus observed probability of the outcome. Analyses will be repeated defining MSKI by type (back pain vs extremity) to evaluate predictive performance for narrowed case definitions.

#### Sample size

We will target equally sized cohorts for Phase I and Phase II. Sample size estimates for Phase II analyses are as described for classification analyses in Phase I cohorts. Based on these estimates we expect these validation cohort sizes to include  > 100 events with  > 100 non-events for each MSKI-specific model. These event counts have been shown to achieve sufficient power in validation studies of logistic regression prediction models [41].

## Discussion

Currently, Military Health System healthcare providers and human performance professionals have limited resources to efficiently and comprehensively identify the factors that influence MSKI risks and outcomes. The PREPARE study will deliver comprehensive clinical assessment batteries that efficiently identify the Service member specific factors that contribute to MSKI risk and undesired post-MSKI outcomes. In the near-term, this will provide contemporary evidenced-based military-relevant, field-expedient MSKI screening and prediction tools to inform Service members’ MSKI susceptibilities and ability to RTD/A following MSKI. In the long-term, this will result in personalized pre- and post-MSKI care, culminating in reduced rehabilitation time and costs and fewer days on limited duty status. Furthermore, the use of semi-automated field-expedient technologies will allow assessments to be immediately translated into practice. PREPARE will provide healthcare providers objective data across the spectrum of MSKI management (prevention to RTD/A) to guide MSKI risk mitigation strategies and make better informed RTD/A decisions; thereby, improving Service member health, medical readiness, and retention within the DoD.

The biggest challenge for completing the proposed work is enrolling a sufficient number of participants to power our analyses. This risk is minimal, as our potential participant population of in-processing active duty Service member at Fort Bragg, NC is 464% larger than is required to power our study. Furthermore, there is the risk that a sufficient number of participants will not sustain a MSKI during our follow-up period; this is unlikely, as MSKIs affect 54–58% of operational Service members [4, 38]. Similarly, we may not enroll a sufficient number of Service members with a MSKI of interest. This risk is also minimal, as our potential patient populations are 276% larger at WRNMMC and 657% larger at Fort Bragg, NC than is required to power our study. Participant attrition and loss to follow-up is mitigated, given our follow-up time points align with routine clinical encounters and outcomes will be collected through the MDR.

In the event the data in the automated reports of the field-expedient assessments provided by the markerless motion capture system and instrumented walkway cannot identify the factors affecting MSKI risks (Specific Aim #1) and post-MSKI outcomes (Specific Aim #2) we can export the raw kinematic and kinetic data. These data may identify minute neuromuscular control deficiencies that are unable to be detected by standard clinical assessments. Additionally, patient-specific demographic data (e.g., sex, age, tobacco use) may be used to increase the robustness of our models [5, 7, 8]. Finally, an alternative analytical approach will establish cut points for each assessment that dichotomize participants into a “at greater than normal risk” or “not greater than normal risk” category [4, 23]. We will then determine if Service members are at greater risk for MSKI or an undesired post-MSKI outcome based on the number of risk factors they possess (i.e., Service members with more risk factors are at greater risk for MSKIs and undesired post-MSKI outcomes) [4].

This study will deliver efficient, comprehensive clinical decision support aids that better enable clinicians to identify Service members at the highest risk of MSKI or post-MSKI undesired outcomes and inform risk mitigation and rehabilitation strategies. The use of semi-automated, field-expedient assessments that require minimal equipment and space will allow the findings of this study to be pushed immediately to clinicians. This will positively impact Service members and healthcare providers as they will have unprecedented access to objective data to guide in-theatre treatment and RTD/A decisions. Personalizing post-MSKI rehabilitative care and making more informed RTD/A decisions will expedite rehabilitation and reintegration and reduce secondary MSKI risk. These benefits will improve Service member health, reduce post-MSKI time to RTD/A, and improve Force medical readiness. Finally, the findings of this study may have translational relevance to other young, active, and otherwise healthy populations.

## Data Availability

The datasets generated and analyzed during the current study are not publicly available because of data sharing restrictions on data generated within the United States Department of Defense; however, data may be available from the corresponding author on reasonable request, following approval from all required regulatory bodies.

## Abbreviations

AUC: Area under the curve
CAT: Computer adaptive testing
DoD: Department of defense
FMS: Functional movement screen
ICD: International classification of diseases
LOESS: Locally estimated scatterplot smoothing
MDR: Military health system data repository
MSKI: Musculoskeletal injuries
NIH: National institutes of health
PROMIS: Patient reported outcomes measurement information system
PREPARE: Pre-neuromusculoskeletal injury risk factor evaluation and Post-neuromusculoskeletal injury assessment for return-to-duty/activity enhancement
RTD/A: return-to-duty/activity
TSK-11: Tampa scale of kinesiophobia
WRNMMC: Walter reed national military medical center
